# Utility of minor determinants in penicillin allergy skin testing

**DOI:** 10.1186/1939-4551-8-S1-A228

**Published:** 2015-04-08

**Authors:** Bob Geng

**Affiliations:** 1 UCLA, USA

## Background

Penicillin allergy is a major concern among patients and physicians. There’s an estimated 5-10% self reported history of penicillin allergy by patients, but vast majority are not true hypersensitivities. Complete penicillin skin test with both major, minor determinants and amoxicillin is the test of choice for diagnosing IgE-mediated penicillin allergy. While Penicilloyl polylysine (major determinant) is widely available, the minor determinants of Penicilloate and Penilloate are not. The goal of this study is to investigate utility of using minor determinants to better detect IgE-mediated penicillin allergy.

## Methods

In this retrospective study, inpatient penicillin skin test records were reviewed from past 6 years from one academic institution. All tests were done with major, minor determinants and amoxicillin along with positive and negative controls. Intradermals were done if prick testing was negative. Number of total positive tests, major determinant only positives, positives to each of the individual minor determinants, and positives to only amoxicillin were obtained.

## Results

Of 528 subjects who underwent inpatient penicillin skin test from 2008-2013, 107 subjects (20.3% positivity) were determined to be positive from skin test based on a wheal >4mm accompanied by flare in setting of appropriate positive and negative controls. Of those positives, 39 subjects (36.4%) reacted to major determinant penicilloyl, and only 13 (12.1%) reacted solely to penicilloyl. 24 subjects (22.4%) reacted to penicillin and 2 (1.9%) only reacted to penicillin. 58 subjects (54.2%) reacted to penicilloate, and 25 (23.4%) only to penicilloate. 23 subjects (21.5%) reacted to penilloate, and 3 (2.8%) only to penilloate. 41 subjects (38.3%) reacted to amoxicillin and 8 (7.5%) only to amoxicillin. There were 41 subjects (38.3%) who only reacted to minor determinants (penicilloate, penilloate and/or penicillin). There were 68 subjects (63.6%) who reacted to a compound other than the major determinant.

## Conclusions

This is one of the largest studies that examined the relative contributions of the various components of penicillin skin test to the detection of IgE-mediated penicillin allergy. While vast majority of administered penicillin is metabolized to the major determinant penicilloyl moiety, it only accounted for a minority of total positive results in our data set. Reactivity only to minor determinants (penicillin, penicilloate and penilloate) and amoxicillin accounted for 63.6% of all the positive results. This study illustrates the utility of minor determinants and the importance of their inclusion in penicillin skin test.

